# Global endometrial transcriptomic profiling: transient immune activation precedes tissue proliferation and repair in healthy beef cows

**DOI:** 10.1186/1471-2164-13-489

**Published:** 2012-09-18

**Authors:** Cathriona Foley, Aspinas Chapwanya, Christopher J Creevey, Fernando Narciandi, Derek Morris, Elaine M Kenny, Paul Cormican, John J Callanan, Cliona O’Farrelly, Kieran G Meade

**Affiliations:** 1 Animal and Bioscience Research Department, Animal & Grassland Research and Innovation Centre, Teagasc, Grange, Co, Meath, Ireland; 2 Comparative Immunology Group, School of Biochemistry and Immunology, Trinity College, Dublin 2, Ireland; 3 Department of Production Animal Studies, Faculty of Veterinary Science, University of Pretoria, Onderstepoort, Pretoria, South Africa; 4 TrinSeq, Trinity Genome Sequencing Laboratory, Institute of Molecular Medicine (IMM), Trinity College Dublin, Dublin 2, Ireland; 5 UCD School of Veterinary Medicine, Dublin 4, Ireland

**Keywords:** Next generation sequencing, Endometrial biopsy, Transcriptome, Immune response, Uterine involution

## Abstract

**Background:**

All cows experience bacterial contamination and tissue injury in the uterus postpartum, instigating a local inflammatory immune response. However mechanisms that control inflammation and achieve a physiologically functioning endometrium, while avoiding disease in the postpartum cow are not succinctly defined. This study aimed to identify novel candidate genes indicative of inflammation resolution during involution in healthy beef cows. Previous histological analysis of the endometrium revealed elevated inflammation 15 days postpartum (DPP) which was significantly decreased by 30 DPP. The current study generated a genome-wide transcriptomic profile of endometrial biopsies from these cows at both time points using mRNA-Seq. The pathway analysis tool GoSeq identified KEGG pathways enriched by significantly differentially expressed genes at both time points. Novel candidate genes associated with inflammatory resolution were subsequently validated in additional postpartum animals using quantitative real-time PCR (qRT-PCR).

**Results:**

mRNA-Seq revealed 1,107 significantly differentially expressed genes, 73 of which were increased 15 DPP and 1,034 were increased 30 DPP. Early postpartum, enriched immune pathways (adjusted *P* < 0.1) included the T cell receptor signalling pathway, graft-versus-host disease and cytokine-cytokine receptor interaction pathways. However 30 DPP, where the majority of genes were differentially expressed, the enrichment (adjusted *P* < 0.1) of tissue repair and proliferative activity pathways was observed. Nineteen candidate genes selected from mRNA-Seq results, were independently assessed by qRT-PCR in additional postpartum cows (5 animals) at both time points. *SAA1/2, GATA2, IGF1, SHC2,* and *SERPINA14* genes were significantly elevated 30 DPP and are functionally associated with tissue repair and the restoration of uterine homeostasis postpartum.

**Conclusions:**

The results of this study reveal an early activation of the immune response which undergoes a temporal functional change toward tissue proliferation and regeneration during endometrial involution in healthy postpartum cows. These molecular changes mirror the activation and resolution of endometrial inflammation during involution previously classified by the degree of neutrophil infiltration. *SAA1/2, GATA2, IGF1, SHC2,* and *SERPINA14* genes may become potential markers for resolution of endometrial inflammation in the postpartum cow.

## Background

The postpartum bovine uterus undergoes involution - a process involving uterine size reduction, contraction, caruncle shedding, necrosis and rejuvenation of endometrial tissue. Involution ensures that the uterus returns to a physiological functioning state, becoming receptive to and supportive of a new conceptus [1]. During involution bovine uteri are invariably exposed to bacterial contamination, clearance and recontamination early postpartum, normally followed by the spontaneous clearance of bacteria 10-15 days postpartum (DPP) in healthy cows [2,3].

The involvement of inflammatory processes and the immune response during involution in the cow has been the subject of numerous recent studies. At a cellular level, uterine inflammation early postpartum is characterised by a neutrophil-rich endometrial inflammatory cell infiltrate [4-6] crucial for acute wound healing [7,8]. Resolution of endometrial inflammation is identifiable in healthy animals by the reduction of the number of neutrophils as involution progresses [5,6,9]. The molecular changes that accompany uterine involution postpartum consists of extensive immune gene activation [10-13]. It is now established that the postpartum endometrial inflammatory response and its allied immune gene activation is a transient feature of the normal physiological events associated with uterine involution [6].

However, a large proportion of cows fail to spontaneously clear bacterial infections postpartum (40%) [14,15] and dysregulation of the immune response in these animals may lead to prolonged inflammation and development of disease [11]. Previously by histopathological assessments of uterine biopsies, we have described endometrial infiltration by leukocytes early during involution which was significantly reduced by 30 DPP. In parallel, we showed significant temporal reduction in toll-like receptor, leukocyte surface receptor, pro-inflammatory and antimicrobial gene transcription during later stages of involution [6]. In the current study we hypothesised that repeat uterine biopsies, at two postpartum time points in healthy cows undergoing involution, would reveal distinct temporal gene expression profiles identifying molecular changes associated with bacterial clearance and the resolution of inflammation.

Recent publications in this area have elucidated the mechanisms of bovine endometrial infection and immune responses in primary cells *in vitro*[16-18]. However, the complexity of the uterine bacterial milieu and immune responses observed *in vivo* are influenced by a variety of animal factors, such as energy balance [19] which cannot be accurately replicated *in vitro*. Therefore, postpartum changes over time, in endometrial biopsies *ex vivo*, are likely to reflect a more accurate profile of the immune response in healthy compared to diseased animals [20]. Interestingly, new approaches have used endometrial explants to characterise endometrial immune function *ex vivo* in response to specific bacterial stimuli [21]. These studies focus on specific gene subsets whereas the current study takes a more comprehensive approach by analysing the entire transcriptome of endometrial biopsies providing a novel and important insight into the regulation of inflammation postpartum.

This is the first study to use next generation sequencing to assess bovine endometrial inflammation postpartum and the temporal resolution of this inflammation during involution in healthy cows. Next-generation sequencing is not limited by the pre-selection of specific candidate genes for absolute gene expression analysis and the digital readout of gene sequence is superior to the relative fluorescence methods used in qRT-PCR and microarray analyses [22]. A genome-wide transcriptomic profile of endometrial biopsies 15 and 30 DPP was generated with mRNA-Seq and subsequently GoSeq KEGG pathway analysis identified enriched gene networks. This data, plus validation of specific gene expression patterns in additional postpartum animals provides evidence for a transcriptomic switch from a pro-inflammatory gene expression phenotype 15 DPP to a tissue regenerative and proliferative phenotype 30 DPP. This dataset highlights the value of large scale genomic approaches toward understanding the immune regulatory networks associated with normal bacterial clearance and the resolution of inflammation in the postpartum cow that may form the basis for future diagnosis of delayed clearance and perturbed immune regulation in diseased animals.

## Results

### mRNA-Seq read alignment and differential gene expression

mRNA-Seq read alignment, summarization and library normalization is tabulated in Additional files 1 (a-c). The average number of raw reads across all samples was 26.79 million and the average number of reads across all samples with one reported alignment was 18.9 million. This data has been deposited in NCBI's Gene Expression Omnibus and are accessible through GEO Series accession number GSE40312.

**Figure 1:**
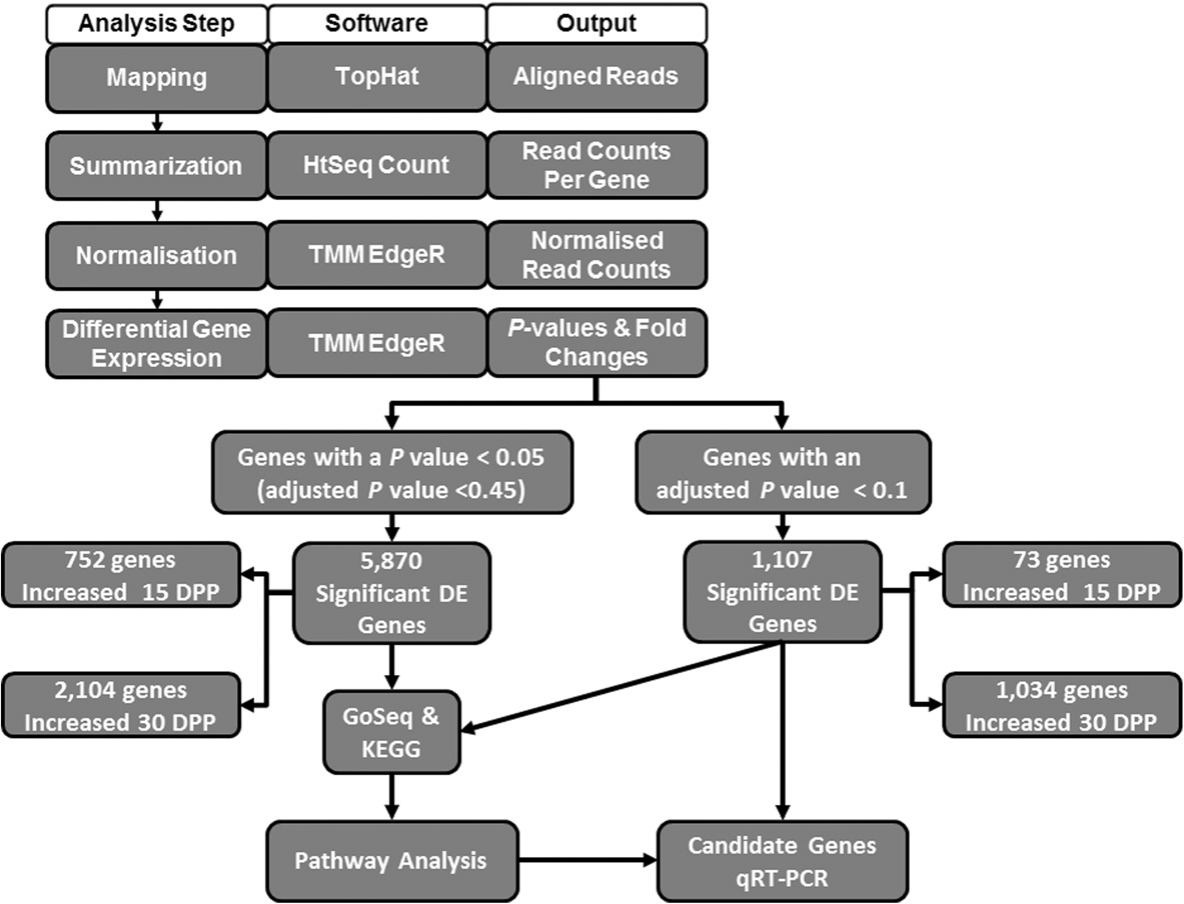
**Work flow for next generation sequencing data analysis, the resulting gene expression output from TMM EdgeR and downstream analysis with differentially expressed genes.** Sequenced reads were mapped to the bovine genome with TopHat, summarised with HtSeq Count and normalised with TMM EdgeR. Output files of differentially expressed (DE) genes with *P* < 0.05 (adjusted *P* < 0.45) and an adjusted *P* < 0.1 were used in GoSeq pathway analysis. Nineteen of the most highly significant DE genes, (adjusted *P* < 0.1) 15 days and 30 days postpartum, were selected for assessment by qRT-PCR in a larger sample set (n = 5).

Two output files were constructed from the EdgeR results, based on different levels of stringency. Using a *P*-value cut off of 0.05, 2,856 significantly differentially expressed genes were identified, of which 752 were elevated 15 DPP and 2,104 elevated 30 DPP. With an adjusted *P-*value of 0.1, the second output file contained 1,107 genes of which 73 were significantly increased 15 DPP and 1,034 significantly increased 30 DPP (Figure 1).

To assess the contribution of changes in endometrial cell subpopulations to the differential gene expression patterns detected between the two post partum time points, the expression of cell surface markers associated with specific leukocyte cell subsets were analysed at a molecular level. The mRNA-Seq data showed that six genes encoding leukocyte cell surface markers *CD11d* (also known as *ITGAD*)*, CD18* (also known as *ITGB2*)*, CD45* (also known as *PTPRC*)*, CD48*, *CD53* and *CD62L* (also known as *SELL*) were significantly elevated at 15 DPP with a *P*-value of < 0.05 but are not significant using an adjusted *P-*value of < 0.1.

### GoSeq KEGG pathway analysis

**Figure 2:**
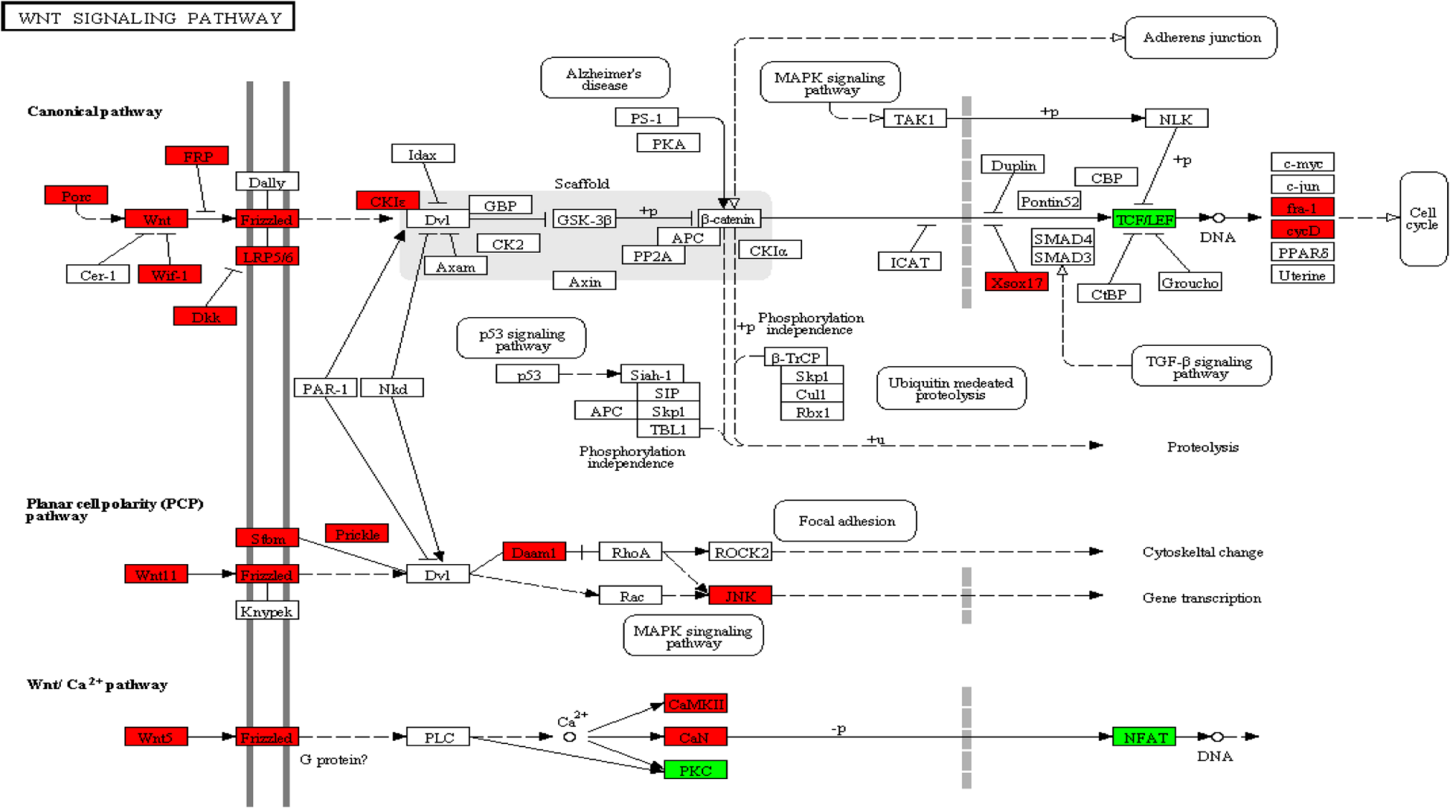
**Differentially expressed genes within the Wnt Signalling pathway.** Genes significantly elevated (*P* < 0.05) 15 DPP are in green and 30 DPP are in red.

**Figure 3:**
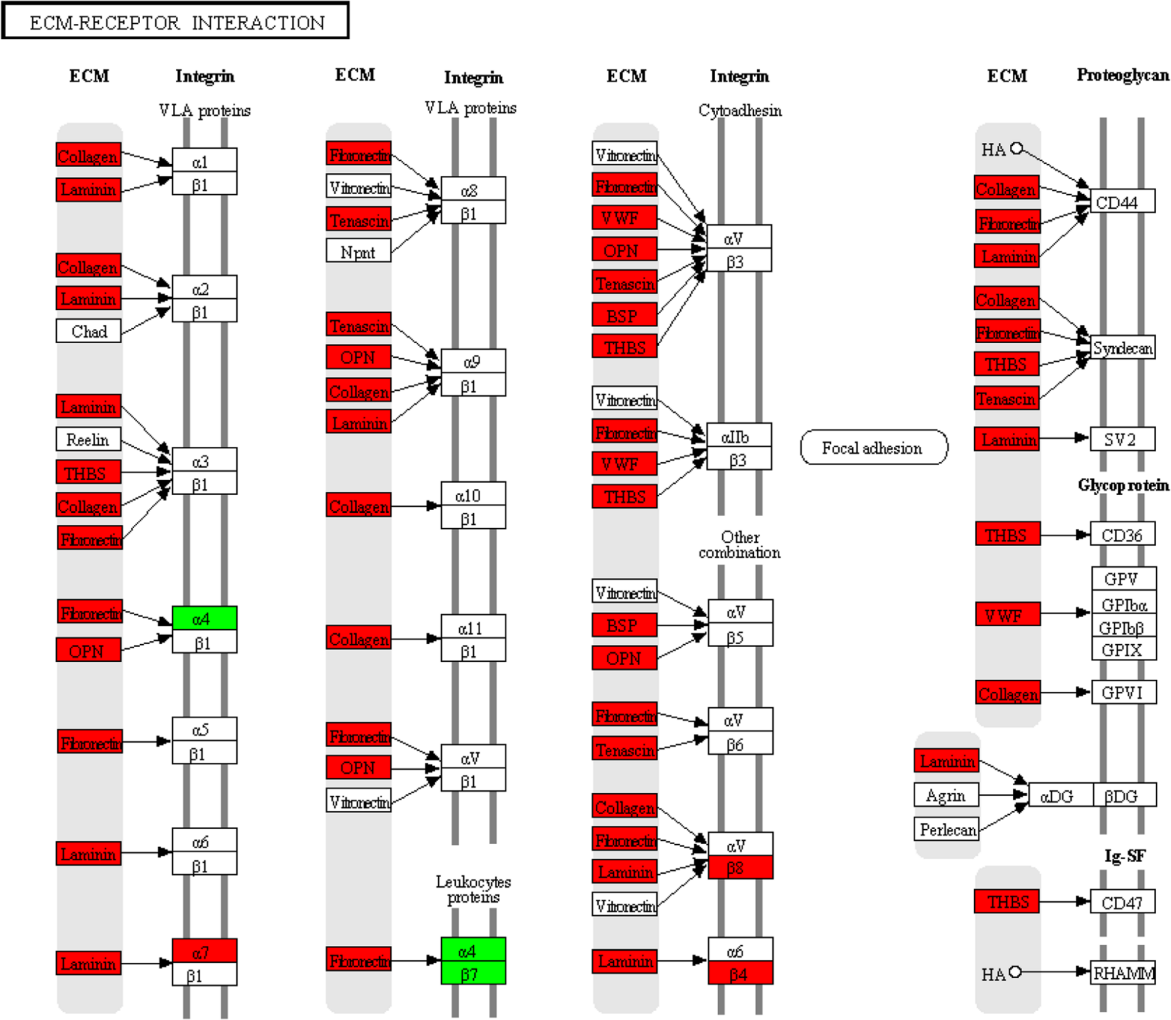
**Differentially expressed genes within the ECM (extra cellular matrix) receptor interaction pathway.** Genes significantly elevated (*P* < 0.05) 15 DPP are in green and 30 DPP are in red.

GoSeq is designed to account for gene length bias and was therefore selected to identify enriched pathways in the dataset. To account for the under representation of bovine genes in GoSeq KEGG pathway analysis genes with a *P* < 0.05 were initially used. Additional analysis was also performed using the more stringent gene set (adjusted *P* of < 0.1) which reduces the risk of false positive genes in GoSeq KEGG pathway analysis. Five pathways were enriched 15 DPP by genes with an adjusted *P* of < 0.1, all of which are also present in the list of enriched pathways containing genes with a *P* < 0.05. Nineteen pathways were enriched 30 DPP containing genes with an adjusted *P* < 0.1 and eighteen of these pathways are also present in the list of enriched pathways containing genes with a *P* < 0.05. The majority of the top enriched pathways (adjusted *P* < 0.1) 15 DPP from both gene datasets are immune associated such as T cell receptor signalling pathway, cytokine-cytokine receptor interaction, natural killer cell mediated cytoxicity, PPAR signalling pathway, rheumatoid arthritis, graft-versus-host disease, allograft rejection and autoimmune thyroid disease (Table 1). A different functional profile is observed 30 DPP depicted by the top enriched pathways (adjusted *P* < 0.1) at this time (Table 2), which are indicative of proliferation and repair such as pathways in cancer, focal adhesion, wingless type (wnt) signalling pathway (Figure 2) and extracellular matrix (ECM)-receptor interaction (Figure 3).

**Table 1:** **Top enriched KEGG pathways (adjusted *P *** **< 0.1) with significantly increased genes 15 days postpartum (*P*** **< 0.05; adjusted *P*** **< 0.1)**

**Enriched KEGG pathways 15 DPP**	***P*-value**	
	**Over represented**	**Under represented**
**Input - Genes with a*P*-value < 0.05**		
Primary immunodeficiency	3.26E-17	1.00E+00
T cell receptor signaling pathway	5.63E-14	1.00E+00
Natural killer cell mediated cytotoxicity	9.16E-14	1.00E+00
Hematopoietic cell lineage	1.90E-13	1.00E+00
Cytokine-cytokine receptor interaction	1.38E-11	1.00E+00
**Input - Genes with an adjusted*P*-value < 0.1**		
PPAR signaling pathway	6.19E-03	1.00E+00
Rheumatoid arthritis	7.63E-03	1.00E+00
Graft-versus-host disease	3.36E-02	9.99E-01
Allograft rejection	3.86E-02	9.99E-01
Autoimmune thyroid disease	4.19E-02	9.99E-01

**Table 2:** **Top enriched KEGG pathways (adjusted *P*** **< 0.1) with significantly increased genes 30 days postpartum (*P*** **< 0.05; adjusted *P*** **< 0.1)**

**Enriched KEGG pathways 30 DPP**	***P*-value**	
	**Over represented**	**Under represented**
**Input - Genes with a*P*-value < 0.05**		
Focal adhesion	4.80E-08	1.00E+00
Axon guidance	4.62E-06	1.00E+00
Hedgehog signaling pathway	4.75E-06	1.00E+00
ECM-receptor interaction	1.07E-05	1.00E+00
Basal cell carcinoma	1.83E-04	1.00E+00
Wnt signaling pathway	2.59E-04	1.00E+00
Arrhythmogenic right ventricular cardiomyopathy (ARVC)	2.60E-04	1.00E+00
Melanogenesis	9.64E-04	1.00E+00
Tight junction	9.69E-04	1.00E+00
Cell adhesion molecules (CAMs)	1.35E-03	9.99E-01
**Input - Genes with an adjusted*P*-value < 0.1**		
Hedgehog signaling pathway	6.03E-08	1.00E+00
Focal adhesion	3.75E-07	1.00E+00
Basal cell carcinoma	1.20E-06	1.00E+00
ECM-receptor interaction	1.01E-05	1.00E+00
Arrhythmogenic right ventricular cardiomyopathy (ARVC)	1.51E-05	1.00E+00
Melanogenesis	1.51E-05	1.00E+00
Wnt signaling pathway	4.60E-05	1.00E+00
Axon guidance	7.34E-05	1.00E+00
Pathways in cancer	8.55E-04	1.00E+00
Protein digestion and absorption	1.24E-03	1.00E+00

### Quantitative expression of candidate genes in additional animals

An extended panel of animals (5 cows) from the original sample set were used for qRT-PCR assessment of 19 candidate genes selected based on a variety of criteria (adjusted *P* < 0.1, read counts, pathway analysis and log_2_ fold change). Thirteen of these genes were annotated in the KEGG pathways: *IGF1* (fold change 7.7)*, MGAT3* (fold change 7.4), *PDGFRA* (fold change 6.8)*, RASGRP2* (fold change 4.7)*, RDH10* (fold change 4.6)*, WNT5A* (fold change 5.9)*, SHC2, BMP6, CTF1, CLDN4* (fold change 8.9)*, CDH5* (fold change 5.9)*, TNFRSF13* (fold change 6.0) and *CD22* (fold change 4.9). The three candidate genes with no pathway annotation in the KEGG database were: *PLAC9* (fold change 6.4)*, GATA2* (fold change 8.4) and *RARRES2* (fold change 6.6). Eleven of these candidate genes (adjusted *P* < 0.1) were represented in the enriched pathways (*P* < 0.05), with the exception of *MGAT3* and *RDH10*.

**Figure 4:**
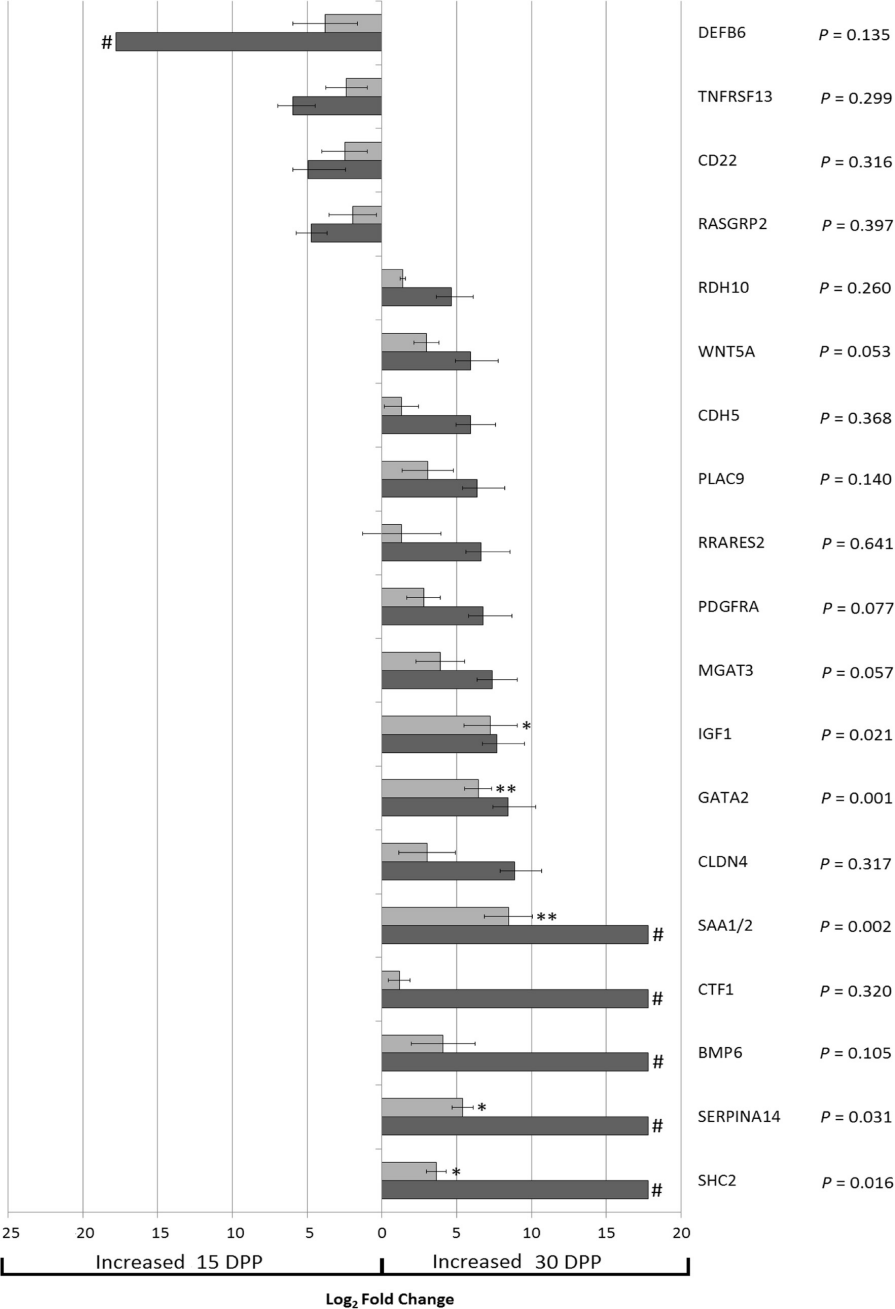
**Log**_**2 **_**fold changes from quantitative real-time PCR and mRNA-Seq for 19 differentially expressed genes in the endometrium of healthy cows between 15 and 30 days postpartum (DPP).** Genes selected were significantly differentially expressed by mRNA-Seq (dark grey bars) (adjusted *P* < 0.1) and the *P*-values on the graph are those for qRT-PCR results (light grey bars) (* = *P* < 0.05, ** = *P* < 0.005). The error bars are representative of the standard error of the mean (SEM). Genes denoted by # have 0 reads at one time point across all samples and for illustrative purposes are represented on this graph as twice the size of the largest Log_2_ fold change. Log_2_ fold changes to the left of 0 are increased 15 DPP and Log_2_ fold changes to the right of 0 are increased 30 DPP.

The qRT-PCR gene expression profile across this gene set corroborated the profile detected using mRNA-seq, with 4 of the 19 genes increased in expression 15 DPP and 15 genes increased in expression 30 DPP for both expression analysis platforms. However the magnitude of the Log_2_ fold change was lower for qRT-PCR compared to mRNA-Seq for the majority of candidate genes which is probably due to the larger sample size used for qRT-PCR introducing greater biological variation (Figure 4).

Of the 19 candidate genes assessed by qRT-PCR, 5 were significantly increased 30 DPP in all 5 additional postpartum animals: Serum Amyloid A - 2 (*SAA1/2*) [*P* = 0.002, log_2_ fold change 8.5], Insulin Growth Factor 1 (*IGF1*) [*P* = 0.021, log_2_ fold change 7.3], GATA binding protein 2 (*GATA2*) [*P* = 0.001, log_2_ fold change 6.4], serpin peptidase inhibitor, clade A (alpha-1 antiproteinase, antitrypsin), member 14 (*SERPINA14*) [*P* = 0.031, log_2_ fold change 5.4] and Src homology 2 domain containing transforming protein C2 (*SHC2*) [*P =* 0.016, log_2_ fold change 3.6] (Figure 4). Other genes were also differentially expressed however these did not achieve statistical significance - *WNT5A* (*P* = 0.053), *PDGFRA* (*P* = 0.077) and *MGAT3* (*P* = 0.057).

Of note, six of the nineteen candidate genes selected from NGS data analysis have 0 reads aligned at one of the time points across all samples. Five genes, *SHC2, SERPINA14, BMP6, CTF1* and *SAA1/2*, have no aligned reads 15 DPP and have an average of 500, 216, 140, 136 and 80 reads respectively, aligned 30 DPP across all animals. One gene (*DEFB6*) has no reads aligned 30 DPP and an average of 28 reads aligned 15 DPP. It is not possible to calculate the fold change in these instances, therefore for illustrative purposes twice the highest Log_2_ fold change was set as the fold change (Figure 4).

## Discussion

Uterine disease and disrupted fertility is the single biggest factor limiting the international dairy industry, with costs estimated at €1.4 billion within the European Union alone [15]. Understanding the role of the immune response in driving bacterial clearance postpartum, but also in restoring homeostasis, is likely to lead to new methods for enhancing fertility.

At both a cellular and a molecular level, there is increasing evidence for an intimate role of the immune response during involution. Neutrophil influx into the endometrium is a common mechanism associated with beneficial bacterial clearance, although evidence suggests that their phagocytic activity may be reduced in disease susceptible animals [23]. Recent research has shown the activation of many immune activation pathways (TLRs) [11-13], inflammatory genes [5], antimicrobial peptides and acute phase proteins [6,10] during the early postpartum period. Expression of the receptor for bacterial lipopolysaccharide, *TLR4* for example, has been shown to be temporally elevated in the postpartum uterus during involution [6,13], between high and low fertility cows [11] and in endometrial epithelial and stromal cells in response to LPS *in vitro*[12].

However, unregulated inflammation can lead to disease [24], therefore a balanced inflammatory immune response is key to sufficient bacterial clearance and the restoration of an endometrial environment capable of supporting a new pregnancy [25]. This resolution of inflammation and the pathways involved in restoration of uterine homeostasis have not been extensively explored in healthy cows. Significantly elevated endometrial inflammatory gene expression (*TNF*, *IL1*, *IL6* and *IL8*) has been previously documented in cows with endometritis at various time points postpartum [20], supporting the hypothesis that dysregulation of the immune response (possibly as a consequence of inadequate bacterial clearance) is a contributory factor to disease development.

Endometrial inflammation was graded based on the number of leukocytes present in the biopsy and candidate gene expression analysis showed significant temporal differences in this limited gene dataset (such as *IL1β, TNF, IFNG, IL8, TAP, DEFB5*) between the two time points across involution, as previously described [6]. The different ontological classes of differentially expressed genes (cytokines, antimicrobial peptides, acute phase proteins) led us to hypothesise that temporal genome wide differential gene expression profiles would shed light on the pathways involved in both the activation of an inflammatory immune response early postpartum but importantly, also on the pathways involved in the regulation of inflammation and the restoration of homeostasis in these healthy cows. In the current study using the same animals, we use next generation sequencing to define the genome wide temporal changes in gene expression at two time points during involution in the postpartum cow.

Approximately only 27% of bovine genes are currently represented in the KEGG database, and therefore using a *P*-value cut-off of < 0.05, 2,856 genes were found to be significantly differentially expressed between endometrial biopsies from the same animals across time points, 15 DPP and 30 DPP. Using increased stringency of an adjusted *P* < 0.1, over 1,100 genes were found to be significantly differentially expressed. In both sets of results, the immune system was predominantly represented by enriched pathways at 15 DPP. These pathways included T cell receptor signalling, cytokine-cytokine receptor interaction, natural killer cell mediated cytotoxicity, graft-versus-host disease and allograft rejection. This result concurs with related studies which have examined the expression of a limited number of candidate immune genes to show the activation of an inflammatory immune response in the postpartum endometrium [5,6,10,11].

However, the majority of the differentially expressed genes detected in this study were significantly increased at the 30 DPP time point. The resolution of inflammation, as defined by histopathological assessment in these cows [6], was supported by the significant decrease in expression of immune pathways as involution proceeds. At this time point (30 DPP) enriched pathways, such as focal adhesion, wnt signalling and ECM-receptor interaction, were associated with tissue regeneration and proliferative activity, reflecting repair processes. This complete temporal change in the endometrial transcriptomic profile from a pro-inflammatory immune response phenotype to a tissue regenerative profile shows rapid but transient immune induction in the uterus to clear bacteria and reduce associated inflammation within a 2 week period in healthy cows. It is likely that changes in both resident and migratory cell populations between time points in response to bacterial infection and during involution account for some of the endometrial transcriptome differences detected. However, expression of a number of leukocyte associated markers (*CD11d, CD14, CD16, CD18, CD45, CD48* and *CD53*) were not significantly different using our stringent selection criteria. Interestingly, *CD62L* - a cell surface adhesion molecule found on the surface of neutrophils was significantly elevated in expression 15 DPP (Log_2_ 4.3 fold, *P* < 0.01), which correlates with the increased neutrophils detected in these animals using histology. However *CD62L* was not significantly differentially expressed using an adjusted *P-*value of < 0.1.

Endometrial receptivity has previously been associated with an increase in extracellular matrix (ECM) remodelling pathways [26,27], which is also one of the top enriched pathways 30 DPP in this study. A cancer associated pathway is another of the top enriched pathways 30 DPP which highlights the proliferative capacity of the endometrium in the current study. Gene-expression patterns linked with the regeneration of damaged tissue closely resemble that of highly malignant tumors, as there is significant enrichment of genes involved in cell proliferation in both instances [28]. In addition to cell proliferative processes, the endometrium is also undergoing regulation of cell growth and differentiation, and tissue homeostasis and repair 30 DPP, evident by the enrichment of the wnt signalling pathway [29,30].

Five candidate genes involved in this temporal transcriptomic change (*SAA1/2, IGF1,GATA2*, *SERPINA14* and *SHC2*), were found to be significantly differentially expressed 30 DPP. The elevation of *SAA1/2* expression 30 DPP, suggests it has a role in postpartum inflammatory resolution in the endometrium of healthy cows. Serum amyloid A (SAA) is an acute phase protein (APP) produced and released by hepatocytes but it is also expressed in extrahepatic bovine tissues [31,32], constitutively in healthy endometria [33,34] and during inflammation [6,10]. Studies have shown that SAA functions to preserve tissue maintenance and homeostasis [35,36] and SAA1/2 in particular has been shown in murine studies to be involved in the provision of immune homeostasis in mucosal tissue [37]. The elevated expression of *SAA1/2* 30 DPP may be indicative of the re-establishment of immune homeostasis later in involution as inflammation resolves.

Results also show the significant increased expression of *GATA2* as involution progresses in the postpartum uterus. Gene expression of interferon-tau (IFN-τ), a luteotrophic molecule, is regulated by the expression of *GATA2* in the bovine trophoblast [38], and is also expressed in endometrial epithelial cells during the peri-attachment period of the conceptus in sheep [39]. *GATA2* has also been recently shown to regulate the gene expression of endomucin, which is critical for cell growth, migration and angiogenesis, to ensure endothelial cell maintenance and physiological function [40].

At systemic and local levels the insulin growth factor (IGF) system is implicated in endometrial repair and healing during involution [41,42]. Decreased expression of *IGF1* has been observed in the previously gravid compared to the non-gravid uterine horn of the same cow 14 DPP [41]. In the present study we compared the progression of involution in the previously gravid uterine horn to a later stage of involution in the same horn and demonstrated that the expression of *IGF1* was elevated 30 DPP. In a murine study it has been shown that bioavailable IGF1 stimulates uteral growth therefore functioning to increase uterine size [43]. A recent bovine study has suggested that an increase of IGF1 bioavailability has a negative effect on oocyte developmental competence [44]. An increase in *IGF1* gene expression 30 DPP in the present study may infer proliferative effects on the uterus.

*SHC2* gene expression was increased 30 DPP and is one of many genes enriching the focal adhesion pathway at this time, suggesting a role for *SHC2* in biological processes such as cell differentiation, motility, regulation of gene expression, cell proliferation, and cell survival in the endometrium later in involution. The Src family kinases are non-receptor tyrosine kinases involved in the mediation of intracellular signal transduction to initiate biological processes such as adhesion, migration, invasion, epithelial-to-mesenchymal transition, angiogenesis, apoptosis resistance and proliferation. Src members are activated by the binding of ligands to either their Src homology 2 (SH2) or 3 (SH3) domains [45]. In bovine endometrial epithelial cells it has been hypothesised that epidermal growth factor receptor may aid in the amplification of oxytocin signalling and activate c-Src resulting in the elevation of prostaglandin F2α production, which is a luteolytic event [46].

Serpin peptidase inhibitor, clade A (alpha-1 antiproteinase, antitrypsin), member 14 (*SERPINA14*) belongs to the serpin superfamily of serine peptidase inhibitors (serpins) [47] and has also been previously called uterine milk protein (UTMP). Serine proteases are associated with immune functions involving inflammation, tissue remodelling, pathogen clearance and apoptosis, the over production of which causes pathologies in auto-immune diseases, tumor metastasis and allergies [48]. Serpins limit the activity of serine proteases thereby regulating the severity of their immune functions [48]. Gene expression of *UTMP* has been observed predominantly in the bovine endometrium, ovary and caruncle tissues and the differential allelic expression of which has been associated with longevity in dairy cattle [49]. Expression of *SERPINA14* has been previously shown to be elevated by estrogens during estrus in cattle [50,51] and also during pregnancy [27]. During pregnancy UTMP is thought to have a role in maternal immune modulation, by inhibiting NK-like activity and thus protecting the conceptus *in utero*[52]. Importantly, another study in sheep suggests that the expression of *UTMP* in the endometrium is a marker of differentiated and functional glandular epithelium [53]. In the present study an increase of *SERPINA14* expression 30 DPP possibly indicates a greater degree of glandular epithelium repair within the endometrium at this time.

Based on the considerable research performed on these genes in other species, our results point toward an important functional role for *SAA1/2*, *GATA2, IGF1, SHC2,* and *SERPINA14* genes, in the restoration of homeostasis during bovine involution.

## Conclusions

Analysis of the entire transcriptome provides a molecular gateway through which the physiological process of involution can be understood in greater detail. It is evident that a pro-inflammatory immune response is instigated early postpartum by the influx of leukocytes into the endometrium, at a cellular level, and by the significant expression of immune genes and pathways 15 DPP, at a molecular level. The transcriptomic response 30 DPP is quite distinct as the majority of differentially expressed genes are increased at this time point, and they represent an enrichment of cellular pathways associated with tissue proliferation and repair.

Therefore, immune activation and inflammation is a transient feature in the healthy postpartum endometrium with a temporal switch toward tissue repair and proliferation pathways that restore homeostasis and prepare the uterus for a subsequent pregnancy. Individual candidate genes involved in this temporal transcriptomic change have been identified as markers of endometrial inflammatory resolution (*SAA1/2*, *GATA2, IGF1, SHC2,* and *SERPINA14)* in cattle*.* Results from this study will form an independent baseline for future studies in animals that develop disease.

## Methods

### Tissue collection, experimental design and total RNA extraction

Endometrial biopsies were collected as part of a previous study 15 and 30 days postpartum (DPP) from 13 mixed breed beef multiparous cows using the Hauptner equine endometrial biopsy instrument. Biopsies were snap frozen in liquid nitrogen and stored at −80°C. Histology analysis of biopsies was used to classify the extent of endometrial inflammation as described previously [6]. In present study the endometrial transcriptomic profiles from endometrial biopsies were assessed by mRNA-Seq (n = 3) and candidate gene expression was measured by qRT-PCR in 5 additional postpartum animals, which included one sample from an animal also used for mRNA-seq.

Frozen endometrial tissue was homogenised and RNA was extracted using 1 ml of Trizol adding 200 μl of chloroform, shaken vigorously and incubated at room temperature for 3 min. Tubes were centrifuged at 12,000 g for 15 min at 4°C. The upper aqueous layer was transferred to another 1.5 ml tube, 500 μl of isopropanol was added and mixed by inverting tube. The sample was incubated at room temperature for 10 min, after which the sample in the 1.5 ml tube was centrifuged at 12,000 g for 10 min at 4°C. The supernatant was removed and the RNA pellet was retained, to which 1 ml or 75% ethanol was added and vortexed to mix. The sample was centrifuged at 10,000 g for 5 min at 4°C, after which the supernatant was removed and the pellet was air dried for 10 min on a heating block at 50°C. The RNA pellet was then reconstituted with 30 μl RNAse-free water, pulse vortexed and placed at −80°C immediately. The NanoDrop ND-1000 UV–vis Spectrophotometer (NanoDrop Technologies Inc., Wilmington, DE, USA) was used to quantify the RNA. The Agilent 2100 Bioanalyser (Agilent Technologies) was used to assess the quality of RNA.

The total RNA, extracted for mRNA-Seq library preparation and qRT-PCR, had an RNA Integrity Number (RIN) which averaged 7.9 ranging between 6.5 – 9 and RNA yields on average of 11.5 μg ranging between 2 – 21 μg. The highest quality total RNA was used as input for the preparation of mRNA-Seq cDNA libraries.

### mRNA-Seq library preparation and next generation sequencing

Initially total RNA was converted into a cDNA sequencing library with the Illumina® mRNA Sequencing Sample Preparation Kit as per manufacturer’s instructions (Protocol: September 2009). Poly-T oligo-attached magnetic beads allowed the isolation and purification of poly-a tailed mRNA from total RNA. Isolated and purified mRNA was fragmented into smaller pieces by divalent cations incubated at 94°C. cDNA synthesis converted mRNA into double stranded cDNA. Overhangs at the end of the fragments were converted into blunt ends with T4 DNA polymerase and Klenow DNA Polymerase. Adenylation of the 3’ ends prepared the fragments for the adapter ligation which allowed fragments to attach to the sequencing flow cell. The purified, size selected cDNA fragments were then subjected to template PCR enrichment and library validation. The resulting sequencing library was quantified using a Qubit® Quantitation Platform and HS dsDNA kit (Invitrogen, Paisley, UK).

The samples were loaded into individual lanes on a flow cell, cluster amplified and sequenced using the Illumina® Genome Analyzer II (GAII) at TrinSeq, the Trinity Genome Sequencing Laboratory, in the Molecular Medicine Institute, Trinity College Dublin (http://www.medicine.tcd.ie/sequencing). The image files created during sequencing of the various fragment clusters were converted into sequence using Illumina Software to achieve 40 bp paired end reads. These reads were then subjected to filtering which removed low-quality sequence and primer contamination. The quality of the reads was assessed using the FastQC software (version 0.4.3) [http://www.bioinformatics.bbsrc.ac.uk/projects/fastqc/]. The assessments used included per base sequence quality, per sequence quality, over represented sequences and per base N count.

### Gene expression data analysis and statistics

Reads were mapped to the bovine genome (version Btau_4.0.62) [54] using the genome aligner TopHat (version 1.3.1) [55,56] and this output was sorted by read name first. Htseq-count [version 0.5.3; http:///www-huber.embl.de/users/anders.HTSeq] was used to summarize the number of aligned reads per exon using the union mode and the Ensembl (version 62) annotation of the bovine genome. Ensembl gene ID’s were used in the output file to identify the number of read counts per gene (Additional files 1(a, b)).

Bioconductor package EdgeR (version 1.6.12) [57] was run within R software (version 2.11.0). To account for biological and technical variation the data were modelled as a negative binomial distribution using a generalisation of the Poisson distribution model. The data were normalised across library size between samples using the trimmed mean of M-values normalization method [58] (Additional file 1c). The Benjamini-Hochberg false discovery rate (FDR) [59] was used in the determination of differential gene expression with an adjusted *P* < 0.1.

### Pathway analysis and statistics

Bovine Ensembl genes were converted to human Ensembl orthologs using the Biomart tool on the Ensembl website (http://www.ensembl.org/biomart/martview). Genes with an adjusted *P* < 0.1 were used in pathway analysis however due to the poor representation of Bovine genes annotated in KEGG pathways, differentially expressed genes with a *P* < 0.05 were also utilised in the downstream pathway analysis. The bioconductor R software (version 2.14.0) package GoSeq (version 1.6.0) [60] pathway analysis tool was implemented to identify pathways enriched by significantly differentially expressed genes (*P* < 0.05 and adjusted *P* < 0.1). Pathways were deemed to be significantly enriched if they had an over represented adjusted *P* < 0.1.

To achieve significantly enriched KEGG pathways in GoSeq, the gene length bias is first quantified by calculating the Probability Weighting Function (PWF) which determines the probability that a gene will be differentially expressed only based on its length. The *P*-values for over-represented (enriched) and under-represented pathways were achieved using the GoSeq default method “Wallenius” by the Wallenius non-central hypergeometric distribution [60]. The advanced KEGG Pathway mapping tool “Search&Colour Pathway” in the KEGG Pathway Database was used to produce graphical images depicting differentially expressed genes (*P* < 0.05) within the enriched pathways.

### Candidate gene selection and primer design

There were 19 candidate genes selected from the results generated by mRNA-Seq differential gene expression and pathway analysis which were subsequently assessed by qRT-PCR. Genes were selected based on a combination of criteria including: an adjusted *P* < 0.1, read counts, pathway analysis and fold change.

The Coding Sequence (CDS) downloaded from Ensembl for each gene and its transcripts were submitted to the UCSC BLAT genome aligner to allow visualisation of exonic sequence separated by intronic sequence http://genome.ucsc.edu/cgi-bin/hgBlat. To ensure coverage across multiple gene transcripts, exons common to all transcripts of the gene were selected for primer design. To predict genomic DNA contamination, primers were designed to be intron spanning. Primers for qRT-PCR were designed using the Primer3 (version 0.4.0) software program [61] to measure expression of the candidate and reference genes. The Basic Local Alignment Search Tool (BLAST) from the National Centre for Biotechnology Information http://www.ncbi.nlm.nih.gov/BLAST/ was used to ensure the specificity of the primers within the bovine genome (Additional file 2).

### cDNA synthesis and quantitative real-time PCR

The High-Capacity cDNA Reverse Transcription Kit was used as per manufacturer’s instructions, (Applied Biosystems, Warrington, UK) to synthesise complementary DNA (cDNA) in a reaction volume of 20 μl with random hexamers by reverse transcribing 0.5 μg of total RNA (sample size of 5 animals, 10 samples 5 x 15 DPP and 5 x 30 DPP). This procedure was repeated with an additional 4 samples (2 x 15 DPP and 2 x 30 DPP) and the resulting cDNA was pooled for use as an interplate calibrator and to achieve the gene efficiencies with a 5-fold dilution series and standard curve.

qRT-PCR reactions were carried out in 96 well plates with a final reaction volume of 20 μl to include: 10 μl SYBR FAST Green Mastermix, 1 μl cDNA, 1 μl of primer mix (forward and reverse primers with a concentration of 250 nM each in the final reaction volume) and 8 μl nuclease free water. Real-time PCR measurements were performed in triplicate to calculate the gene efficiencies and in duplicate to measure the gene expression levels in each sample. The Applied Biosystems Fast 7500 (version 2.0.1) instrument was used with the following cycling parameters: 95°C for 20 sec; 40 cycles of 95°C for 3 sec; 60°C for 30 sec, followed by amplicon dissociation (95°C for 15 sec; 60°C for 60 sec; 95°C for 15 sec and 60°C for 15 sec). Disassociation curves were examined for the presence of a single PCR product for each gene amplified. A non-template control (NTC) was included in each plate.

### Quantitative real-time PCR data analysis and statistics

The reaction efficiencies for 19 primer sets using the pooled cDNA sample were calculated using a 5-fold dilution series to generate a standard curve. Triplicate samples for each cDNA dilution in the series were used and the average raw cycle threshold (Ct) value from the qRT-PCR reaction was then calculated. The standard curve was constructed and the efficiency of the reaction was calculated with the formula; 10(−1/m)-1 where m is the slope of the line. An acceptable efficiency (E) was between 0.9 and 1.1. The efficiency correction was then carried out on the average raw cycle threshold (Ct) values, from the 10 test samples (1 in 5 dilution of cDNA from each cow) in duplicate, using the software package GenEx 5.2.1.3 (MultiD Analyses AB, Gothenburg, Sweden). Variation in PCR efficiency was calculated by the GenEx software. A multitude of plates were used and to account for inter-plate variation, pooled cDNA acted as an inter-plate calibrator (IPC) and the gene ACTB was amplified on each plate in triplicate. The software package GenEx was used to normalise all samples for the inter-plate variation.

Relative gene expression levels were determined with the use of highly stable reference genes. The stability of 5 different reference genes was examined across all samples using qRT-PCR. The reference genes included glyceraldehyde 3-phosphate dehydrogenase (*GAPDH*), β-actin (*ACTB*), suppressor of zeste 12 homolog (*SUZ12*), zinc finger protein 131 (*ZNF131*) and ribosomal protein S9 (*RPS9*) [62,63]. The data were analyzed using geNorm ([64]; accessible through MultiD Analyses AB, Gothenburg, Sweden) to measure the overall stability of the reference genes under examination. Results indicated that *GAPDH* and *ACTB* were the most stable reference genes and had a stability value (M-value) of 0.6, and were chosen as the reference genes for subsequent data analysis. The GenEx 5.2.1.3 software program was used to carry out pair-wise normalisation of the expression values for test samples in the 15 DPP group referenced to their respective expression values at 30 DPP. Following this the expression values were converted to Log_2_ scale. Significant differences between 15 DPP and 30 DPP sample groups were analysed using a Student’s t-test with the GraphPad Prism version 3.0 software package on the normalised Ct values.

## Competing interests

The authors declare that they have no competing interests.

## Authors’ contributions

CF performed data analysis and wrote the manuscript. KGM and COF designed the animal study, interpreted the data, prepared and edited the manuscript. AC performed animal health examinations, collected the endometrial biopsies and histological classification of all biopsies under the guidance of JJC. FN and KGM assisted with sample collection. DK, EK and PC provided valuable guidance during mRNA-Seq library preparations and performed the sequencing. CJC performed expert bioinformatics analysis of NGS data. All authors read and approved the final manuscript.

## Supplementary Material

Additional file 1 **a: Alignment of reads to the bovine genome with TopHat.** TopHat calls the Bowtie software to align reads to the bovine genome. This table shows the combined Bowtie output from paired end reads for all samples that are reported in the “logs” output from TopHat. b: Summarization of read counts for each gene with HtSeq-Count. The numbers of reads representing genes annotated in Ensembl and subsequently filtered for downstream analysis. c: Normalised library sizes with TMM-EdgeR. The number of reads used for each animal is shown before and after normalisation using TMM-EdgeR. Click here for file

Additional file 2 **Primer details for candidate and reference genes used in real-time RT-qPCR.** ACTB and GAPDH were the reference genes used to calculate relative fold changes for all other candidate genes. Click here for file
