# Supplementary material for: Global endometrial transcriptomic profiling: transient immune activation precedes tissue proliferation and repair in healthy beef cows
Source: BMC Genomics. 2012 Sep 18;13:489. doi: 10.1186/1471-2164-13-489 (PMC3544567; doi:10.1186/1471-2164-13-489)
Supplement: Additional file 1 — a: Alignment of reads to the bovine genome with TopHat. TopHat calls the Bowtie software to align reads to the bovine genome. This table shows the combined Bowtie output from paired end reads for all samples that are reported in the “logs” output from TopHat. b: Summarization of read counts for each gene with HtSeq-Count. The numbers of reads representing genes annotated in Ensembl and subsequently filtered for downstream analysis. c: Normalised library sizes with TMM-EdgeR. The number of reads used for each animal is shown before and after normalisation using TMM-EdgeR. [file 1471-2164-13-489-S1.doc]

Table 1: Top enriched KEGG pathways (*P*<0.01) with significantly increased genes 15 days postpartum (*P*<0.05; adjusted *P*<0.1).

| **Enriched KEGG Pathways 15 DPP** | ***P*-value** | |
| --- | --- | --- |
|  | **Over Represented** | **Under Represented** |
| **Input - Genes with a *P*-value < 0.05** | | |
| Primary immunodeficiency | 3.26E-17 | 1.00E+00 |
| T cell receptor signaling pathway | 5.63E-14 | 1.00E+00 |
| Natural killer cell mediated cytotoxicity | 9.16E-14 | 1.00E+00 |
| Hematopoietic cell lineage | 1.90E-13 | 1.00E+00 |
| Cytokine-cytokine receptor interaction | 1.38E-11 | 1.00E+00 |
| **Input - Genes with an adjusted *P*-value < 0.1** | | |
| PPAR signaling pathway | 6.19E-03 | 1.00E+00 |
| Rheumatoid arthritis | 7.63E-03 | 1.00E+00 |
| Graft-versus-host disease | 3.36E-02 | 9.99E-01 |
| Allograft rejection | 3.86E-02 | 9.99E-01 |
| Autoimmune thyroid disease | 4.19E-02 | 9.99E-01 |
