# Supplementary material for: Global endometrial transcriptomic profiling: transient immune activation precedes tissue proliferation and repair in healthy beef cows
Source: BMC Genomics. 2012 Sep 18;13:489. doi: 10.1186/1471-2164-13-489 (PMC3544567; doi:10.1186/1471-2164-13-489)
Supplement: Additional file 2 — Primer details for candidate and reference genes used in real-time RT-qPCR. ACTB and GAPDH were the reference genes used to calculate relative fold changes for all other candidate genes. [file 1471-2164-13-489-S2.doc]

Table 2: Top enriched KEGG pathways (*P*<0.01) with significantly increased genes 30 days postpartum (*P*<0.05; adjusted *P*<0.1).

| **Enriched KEGG Pathways 30 DPP** | ***P*-value** | |
| --- | --- | --- |
|  | **Over Represented** | **Under Represented** |
| **Input - Genes with a *P*-value < 0.05** | | |
| Focal adhesion | 4.80E-08 | 1.00E+00 |
| Axon guidance | 4.62E-06 | 1.00E+00 |
| Hedgehog signaling pathway | 4.75E-06 | 1.00E+00 |
| ECM-receptor interaction | 1.07E-05 | 1.00E+00 |
| Basal cell carcinoma | 1.83E-04 | 1.00E+00 |
| Wnt signaling pathway | 2.59E-04 | 1.00E+00 |
| Arrhythmogenic right ventricular cardiomyopathy (ARVC) | 2.60E-04 | 1.00E+00 |
| Melanogenesis | 9.64E-04 | 1.00E+00 |
| Tight junction | 9.69E-04 | 1.00E+00 |
| Cell adhesion molecules (CAMs) | 1.35E-03 | 9.99E-01 |
| **Input - Genes with an adjusted *P*-value < 0.1** | | |
| Hedgehog signaling pathway | 6.03E-08 | 1.00E+00 |
| Focal adhesion | 3.75E-07 | 1.00E+00 |
| Basal cell carcinoma | 1.20E-06 | 1.00E+00 |
| ECM-receptor interaction | 1.01E-05 | 1.00E+00 |
| Arrhythmogenic right ventricular cardiomyopathy (ARVC) | 1.51E-05 | 1.00E+00 |
| Melanogenesis | 1.51E-05 | 1.00E+00 |
| Wnt signaling pathway | 4.60E-05 | 1.00E+00 |
| Axon guidance | 7.34E-05 | 1.00E+00 |
| Pathways in cancer | 8.55E-04 | 1.00E+00 |
| Protein digestion and absorption | 1.24E-03 | 1.00E+00 |
